# From Sequence to Solution: Intelligent Learning Engine Optimization in Drug Discovery and Protein Analysis

**DOI:** 10.3390/biotech13030033

**Published:** 2024-09-01

**Authors:** Jamal Raiyn, Adam Rayan, Saleh Abu-Lafi, Anwar Rayan

**Affiliations:** 1 Computer Science Department, Faculty of Science, Al-Qasemi Academic College, Baka EL-Garbiah 30100, Israel; raiyn@qsm.ac.il; 2 NGS Ac-Tech—Next Generation Scholars Ltd., Kabul 2496300, Israel; adam.rayan67@gmail.com; 3 Faculty of Pharmacy, Al-Quds University, Abu-Dies 144, Palestine; sabulafi@staff.alquds.edu; 4 Science and Technology Department, Faculty of Science, Al-Qasemi Academic College, Baka EL-Garbiah 30100, Israel

**Keywords:** optimization, intelligent learning engine, modeling, protein analysis, classification

## Abstract

This study introduces the intelligent learning engine (ILE) optimization technology, a novel approach designed to revolutionize screening processes in bioinformatics, cheminformatics, and a range of other scientific fields. By focusing on the efficient and precise identification of candidates with desirable characteristics, the ILE technology marks a significant leap forward in addressing the complexities of candidate selection in drug discovery, protein classification, and beyond. The study’s primary objective is to address the challenges associated with optimizing screening processes to efficiently select candidates across various fields, including drug discovery and protein classification. The methodology employed involves a detailed algorithmic process that includes dataset preparation, encoding of protein sequences, sensor nucleation, and optimization, culminating in the empirical evaluation of molecular activity indexing, homology-based modeling, and classification of proteins such as G-protein-coupled receptors. This process showcases the method’s success in multiple sequence alignment, protein identification, and classification. Key results demonstrate the ILE’s superior accuracy in protein classification and virtual high-throughput screening, with a notable breakthrough in drug development for assessing drug-induced long QT syndrome risks through hERG potassium channel interaction analysis. The technology showcased exceptional results in the formulation and evaluation of novel cancer drug candidates, highlighting its potential for significant advancements in pharmaceutical innovations. The findings underline the ILE optimization technology as a transformative tool in screening processes due to its proven effectiveness and broad applicability across various domains. This breakthrough contributes substantially to the fields of systems optimization and holds promise for diverse applications, enhancing the process of selecting candidate molecules with target properties and advancing drug discovery, protein classification, and modeling.

## 1. Introduction

In the ever-evolving landscape of science and technology, the critical task of accurately identifying and optimizing potential candidate molecules from a vast array of options is of the utmost importance [1]. This process is especially significant in the life sciences, where precise selection is key to advancing drug discovery [2], protein classification and modeling [3], and other areas in bioinformatics [4] and cheminformatics [5]. This paper introduces an innovative approach, “intelligent learning engine (ILE) optimization technology,” which is designed to revolutionize and optimize the screening process.

The primary aim of this paper is to address the challenges to effectively optimizing screening processes, focusing on the efficient selection of candidates with desirable characteristics. While the applications of this technology are broad, spanning various scientific and technological fields, this paper primarily underscores its utility in bioinformatics and cheminformatics. However, it is important to note the wide applicability of this advanced optimization technology in diverse domains, such as healthcare, finance, telecommunications, and so forth.

Across different scientific and technological areas, the recurring challenge is selecting the most appropriate candidate molecules from a vast array of possibilities. To tackle this challenge, various approaches have been developed, categorized primarily into optimization methods and modeling techniques. The key optimization methods include the following: 1—genetic algorithms (GAs): these methods generate and evaluate a population of potential solutions [6,7,8]; 2—simulated annealing (SA): a global optimization method utilizing a stochastic acceptance function [9,10]; 3—taboo searching (TBS): this approach combines extensive exploration with targeted local searches [11]; 4—stochastic elimination (ISE): this improves the efficiency of stochastic programming by eliminating suboptimal solutions early in the process [12,13,14]; 5—statistical methods (SMs): these methods optimize objective functions using Bayesian models [15,16]. The key modeling methods include the following: 1—neural networks (NNs): brain-inspired computational models [17,18,19]; 2—support vector machines (SVMs): these use statistical learning theory to classify data [20,21,22]; 3—Monte Carlo (MC) methods: these generate random samples for stochastic algorithms [23,24]; 4—Bayesian networks and Markov models: these offer probabilistic frameworks for optimization, aiding in decision making and learning from data [25,26]; 5—hidden Markov models (HMMs): these are effective for modeling time-series and linear sequences [27,28]; 6—discriminant analysis (DA): this uses statistical variables to classify objects based on their properties [29,30].

The diversity of these methods reflects the complexity of optimization challenges across various fields. It highlights the ongoing need for innovative approaches to select candidates with target properties, a gap this paper aims to fill with the introduction of ILE optimization technology. In the realm of pharmaceutical development, indexing, the efficient cataloging and analysis of molecules, is a pivotal process [2]. It organizes molecules based on specific characteristics and streamlines the retrieval, analysis, and comparison of molecular data [31]. A pressing concern in this area is the increasing use of non-antiarrhythmic drugs, such as antihistamines [32,33,34] and antipsychotics [35,36], which are implicated in prolonging the QT interval [37,38] and thereby increasing the risk of drug-induced long QT syndrome (LQTS)—a condition marked by potentially fatal cardiac arrhythmias.

Central to understanding LQTS is the human ether-à-go-go-related gene (hERG) potassium channel [39,40], instrumental in cardiac excitability and rhythm regulation. The hERG channel’s unique structure makes it susceptible to interactions with a diverse array of drugs, leading to QT interval prolongation—a phenomenon termed “acquired LQTS”. Over 80% of drugs that prolong the QT interval are known to inhibit the hERG K+ channel [12]. This binding, often weak and reversible, poses a significant risk, as it can escalate to torsades de pointes (TdP), a severe form of ventricular tachycardia, even in patients with otherwise normal hearts.

Given these risks, assessing TdP liability is a critical step in drug development, typically evaluated through the drug’s binding affinity to the hERG channel. The advent of ILE technology marks a transformative step in this regard. This chemoinformatics approach, utilizing descriptors such as molecular weight, logP, and the number of rotatable bonds, makes it possible to differentiate between hERG potassium channel blockers and non-blockers. The ILE model assigns a hERG liability index (ELI) to each molecule, estimating its potential as a hERG channel blocker. Such advanced in silico methods are invaluable for early-stage toxicity assessment, enhancing the safety and efficacy of drug development. ILE technology, therefore, represents a significant leap forward in mitigating hERG-related liabilities, paving the way for safer pharmaceutical innovations.

ILE optimization technology, a cornerstone of this study, has already demonstrated groundbreaking potential in drug development. Notably, it has played a pivotal role in the formulation and evaluation of several cancer treatment candidates. For instance, IDD-1040, a paclitaxel–lipoate conjugate, exhibited remarkable anti-tumor efficacy in non-small-cell lung cancer models, outperforming conventional treatments in both tumor inhibition and toxicity profiles [41]. Similarly, IDD-1010, a novel docetaxel–biotin conjugate, proved to be superior to traditional drugs in treating prostate cancer and demonstrated enhanced therapeutic effectiveness and safety [42]. These instances underscore the transformative impact of ILE optimization technology on pharmaceutical innovation, setting a promising precedent for its application in protein analysis and other scientific domains.

Previous studies on protein identification and classification have employed a variety of techniques, including support vector machines (SVMs) [43], hidden Markov models (HMMs) [44], neural networks [45], and probabilistic classifiers [46]. These foundational methods have paved the way for more advanced approaches, such as the ILE technology presented in this study, which notably enhances the accuracy and efficiency of protein analysis.

To summarize, this study introduces an advanced technique designed to significantly enhance screening processes, which has demonstrated its versatility across a wide spectrum of applications, ranging from the selection of drug candidates to the categorization of protein families and beyond. ILE optimization technology transcends the traditional boundaries of bioinformatics and cheminformatics and shows immense potential in various fields. Its capabilities include identifying individuals with a high risk of disease, optimizing investment strategies, improving resource allocation in communication networks, and navigating complex transportation systems, among other uses.

While the primary focus of this study was the application of this technology in bioinformatics, our empirical evaluations centered on several key areas. These included molecular activity indexing; the classification of proteins, such as G-protein-coupled receptors; and the homology-based modeling of serine proteases. These examples highlight the broad impact of ILE optimization technology and illustrate its potential to revolutionize screening processes across diverse scientific and technological fields.

## 2. Materials and Methods

This study utilizes the intelligent learning engine (ILE), a novel optimization algorithm for classifying objects and indexing chemicals for their activity against biological targets.

**Figure 1:**
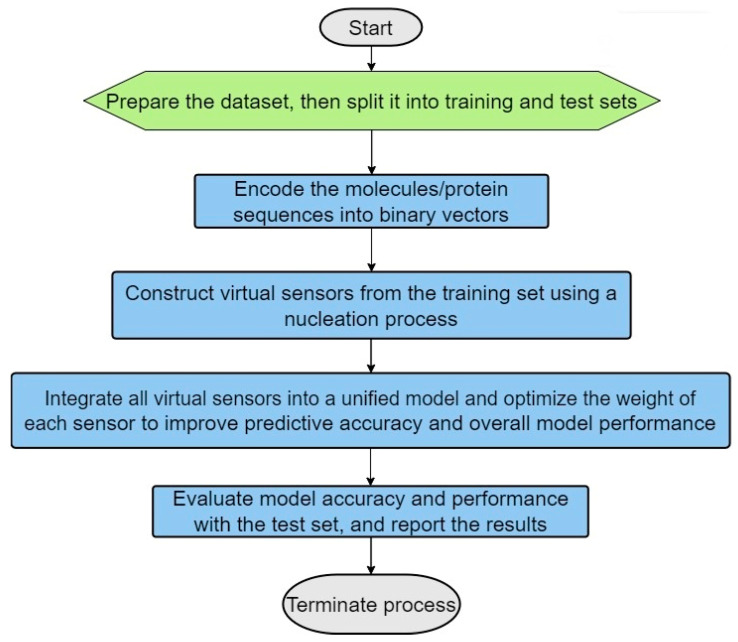
Primary steps involved in the ILE (iterative learning estimation) algorithm.

**Figure 2:**
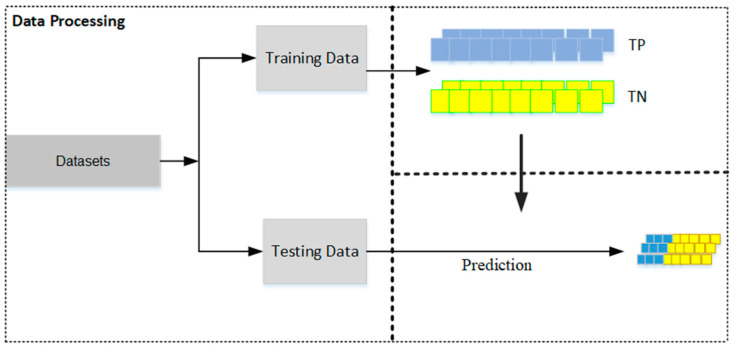
The dataset is partitioned into a training set, used to construct prediction models, and a testing set, used to evaluate the performance of the models.

The following are the main steps in the process (see the algorithm flowchart in Figure 1):

(1) Dataset Preparation: Two distinct datasets are prepared—one containing true positive (TP) matches and the other true negative (TN) matches. These datasets should then be split into training and testing sets, with a typical allocation of two-thirds for training and one-third for testing (see Figure 2). This division is crucial for facilitating effective model training and validation.
(2) Encoding of the Molecules/Protein Sequences into Binary Vectors: The molecules’ protein sequences are encoded into binary vectors, such that each position along the vector is marked as 1 if it has the specific characteristic (for example if its molecular weight falls in a range of 155 to 220 daltons or if the amino acid type at a certain position is aspartic acid), and otherwise as 0.
(3) Virtual Sensor Construction through Nucleation: Virtual sensors are defined by their sensor weight scores (SWSs), which are determined for specific segments of the binary vector. To calculate a sensor’s overall SWS, logical operations such as exclusive OR (XOR) and exclusive NOR (XNOR) are utilized to integrate the sensors with segments of the vector. This facilitates the dynamic generation of features capable of identifying distinct patterns in the binary vectors, especially patterns that mirror the intrinsic biological or chemical attributes of the molecules or proteins involved.
(4) Sensor Optimization: The configuration of sensors is optimized using scoring functions such as specificity, sensitivity, and the Matthews correlation coefficient (MCC). The optimized sensors are then evaluated against the test set to verify their accuracy and reliability; this is aimed at minimizing false positives and false negatives.
(5) Maximization of Virtual Sensor Efficiency: Factors are applied to the weights of the virtual sensors to boost their effectiveness and to augment the model’s capability to distinguish between true positive (TP) and true negative (TN) cases.
(6) Usage of the Optimized Selected Sensors for Modeling Purposes: The refined model, which includes the selected optimized virtual sensors, is applied to specific tasks, such as indexing molecular activity, identifying and classifying proteins, modeling homology, and so forth.

## 3. Building Better Virtual Sensors: An Iterative Strategy of Nucleation and Updates

Virtual sensors are integral to our algorithm, leveraging sensor weight scores (SWSs) to discern the discriminative power of binary vector segments in distinguishing between positives and negatives. These portions correspond to fragments within dataset items or queries, with SWSs being computed based on sensor scoring rules (SSRs), which are adapted to suit true positive (TP) and true negative (TN) items differently. For protein sequences, this process involves segmenting the sequences into frames, each frame representing a distinct subset of amino acids. The SWSs for these frames are then calculated within the training set, facilitating the creation of virtual sensors. The process of sensor selection and replacement (SSR) employs logical operations such as XOR (for TN items) and XNOR (for TP items), which are used to integrate sensors with vector segments, thereby computing the SWSs for these segments. The aggregated SWSs of these segments contribute to the overall sensor score.

**Figure 3:**
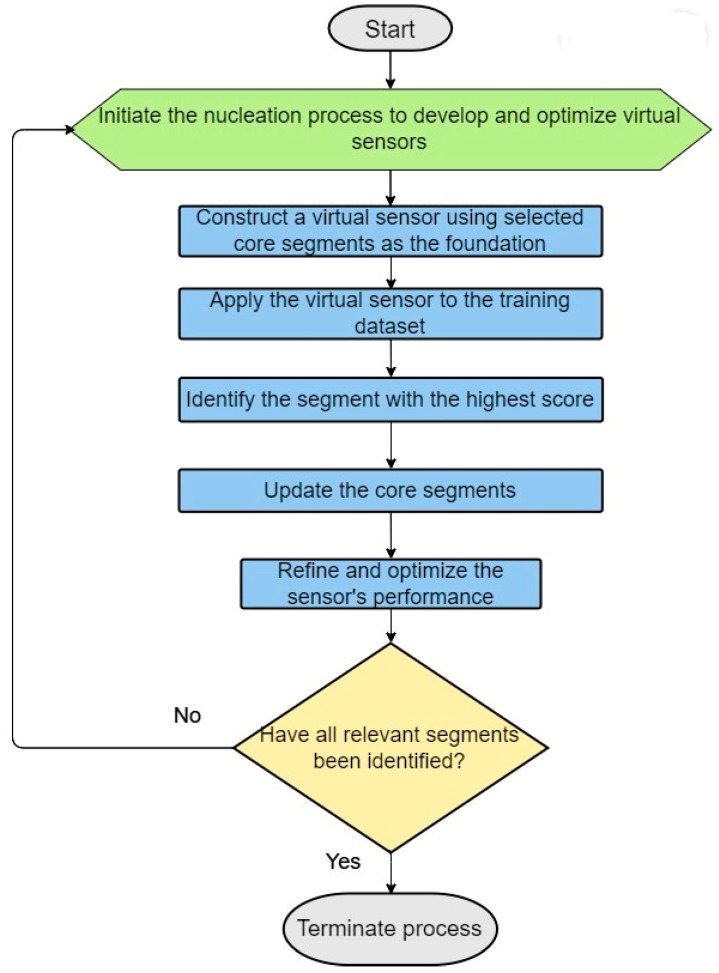
Steps in the dynamic construction of virtual sensors employing nucleation and iterative updates.

Building on this foundation, this article delves into the iterative strategy behind constructing more effective virtual sensors, as outlined in our case study for step 2.3 of the ILE algorithm (see Figure 3). This begins with developing a virtual sensor specifically designed to predict the likelihood of a segment’s being the initial transmembrane helix, based on a comprehensive analysis rule set derived from core segments identified in the training dataset. Selection of the initial core segments is based on experimental data and/or expert insights, with a focus on the physicochemical and relational attributes of segment positions.

This virtual sensor is then iteratively applied across the training set to pinpoint likely segments that represent the first transmembrane helix, incorporating the highest scoring segment into the core for subsequent virtual sensor development. This cyclical enhancement continues until a refined sensor emerges, capable of accurately detecting the first transmembrane helix.

Critical to our methodology is the development of virtual sensors for all seven transmembrane helices, ensuring that each G protein-coupled receptor (GPCR) in the positive set is thoroughly screened for the presence and proper sequence of these helices. This guarantees a robust dataset. We calculate an overall score for each GPCR based on the combined scores from the seven virtual sensors, fine-tuning the weight of each sensor to maximize predictive efficiency and performance.

Through this systematic and iterative approach, we significantly advance the development of virtual sensors, enhancing their accuracy and efficiency at identifying essential transmembrane helix segments. Such progress is crucial for protein classification, homology-based modeling, and deepening our understanding of GPCR structures and functions.

The molecular descriptors used to model hERG liability were calculated with Molecular Operating Environment (MOE) software, version 2009.10, available at http://www.chemcomp.com (accessed on 15 February 2017). These descriptors include a variety of properties, such as molecular weight, log P, hydrogen-bond donors and acceptors, solubility, total molecular charge, the distribution of the molecular charge, the types and numbers of atoms within the molecule, topological indices, connectivity indices such as Chi indices, and the number of aromatic rings [47]. To evaluate and confirm the model’s accuracy in predicting hERG liability, the dataset containing both active and inactive ligands was divided, allocating two-thirds for training and one-third for testing. The division of these sets was carried out using a random selection module that was developed in-house.

## 4. Results

To address the increasing complexity of managing vast amounts of data from newly sequenced proteins and DNA, innovative computational methods are essential. These methods are designed to establish links between sequences and functions (commonly known as classification) and to predict 3D structures from sequences. In this paper, we demonstrate our approach by explaining how we determined whether a given protein belonged to the GPCR family and highlight the utility of this approach for identifying and classifying proteins (ICP).

Protein identification and classification and multiple sequence alignment involve significant computational challenges. These tasks are known to be nondeterministic polynomial-time hard (NP-hard), such that the number of potential solutions increases exponentially with the length of the amino acid sequence or the number of residues in a moiety.

Our study analyzed a total of 167 proteins, which included 31 acetylcholine receptors, 44 adrenoreceptors, 38 dopamine receptors, and 54 serotonin receptors. The outcomes were benchmarked against two established methods in this field, the covariant-discrimination algorithm [48] and the support vector machine algorithm, tailored for amine receptor classification [49].

Using the first method, the true positive (TP) classification rates for the acetylcholine, adrenoreceptor, dopamine, and serotonin items were 67.74%, 88.64%, 81.58%, and 88.89%, respectively, leading to an overall accuracy of 83.23%. The second method was more accurate, correctly classifying 93.6% of the TP acetylcholine items, 100.00% of the TP adrenoreceptor items, 92.1% of the TP dopamine items, and 98.2% of the TP serotonin items, resulting in an overall accuracy of 96.4%.

Remarkably, our algorithm, the ILE, achieved perfect accuracy, classifying 100% of the TP items associated with the acetylcholine, adrenoreceptor, dopamine, and serotonin receptors. This translates to an overall accuracy of 100%. Table 1 presents the results of our virtual high-throughput screening, comparing the performance of the established algorithms with the ILE method in identifying GPCR proteins.

Table 2 shows the results from a virtual high-throughput screening, showcasing the efficacy of various established algorithms for categorizing GPCR proteins into their respective superfamilies, A, B, or C. For purposes of comparison, these outcomes are juxtaposed with the performance of the ILE method.

Table 3 presents the results of a virtual high-throughput screening, depicting the classification of GPCR proteins at their initial subfamily level—amine, peptide, and olfactory—using various well-known algorithms. These results are compared with the performance of the ILE method.

Table 4 presents the results from a virtual high-throughput screening, demonstrating the classification of GPCR proteins at their secondary subfamily level into adrenergic, dopamine, and histamine categories. The classifications, achieved using various well-recognized algorithms, are compared with the performance of the ILE method.

Reviewing Table 1, Table 2, Table 3 and Table 4 clearly demonstrates that the application of ILE technology in protein classification offers notable improvements over other methods such as SVMs [43] and HMMs [44], which have faced limitations and required performance enhancements, particularly in specificity, selectivity, and overall accuracy. The advancements offered by ILE, especially in accurately classifying proteins, highlight its potential to replace or complement existing technologies [45,46].

Homology Modeling: Achieving precise multiple sequence alignment (MSA) is essential for improving the accuracy of pairwise sequence alignments, reducing misalignments, and enabling the creation of more reliable 3D models. The ILE method is particularly advantageous when working with protein families that share a common fold and include multiple members, as it allows for the effective interpretation of sequences in the database. This facilitates accurate MSA and the construction of optimal comparative models.

To assess the ILE method’s effectiveness, we analyzed 124 unique proteins from the serine protease family, sourced from the Brookhaven Protein Data Bank (PDB). We calculated the sequence identity score for each pair and employed the ILE method to optimize sequence alignment. This MSA process identified residues in 98 of the proteins, while 28 proteins had incomplete coordinates for at least one residue in their experimentally determined 3D structures. We extracted the alpha carbons (Cα) of residues from selected PDB structures and performed structural superimposition. The quality of the models was evaluated by comparing superimpositions of our homology-based models on X-ray structures of the proteins and by measuring the Cα root-mean-square deviations (RMSDs).

Table 5 presents the results of our homology modeling study, which demonstrate the efficacy of the ILE method across various target–template identity classes within the serine protease family.

The multiple sequence alignment (MSA) matrix, generated using the ILE method on a dataset of serine proteases, was utilized to identify sequence segments suitable for comparative modeling. This process involved a voting approach, where the contribution of each amino acid conservation at a given sequence position was determined based on its frequency at that position, as calculated by Equation (1). These frequency measurements were performed for all the sequences in the dataset.

(1) $$Cij=\frac{nij}{k}\times 100\%$$

In this context, *Cij* represents the conservation factor for residue type *i* at sequence position *j*. The term *nij* denotes the number of sequences containing amino acid *i* at position *j* in the multiple sequence alignment, while *k* refers to the total number of sequences in the dataset. A positional conservation threshold (PCT) was established to indicate whether the conservation factor for residue type *i* at sequence position *j*, as per Equation (1), was above a certain point. Demarcating a PCT in refining models is advantageous, as it leads to the development of more accurate homology-based models.

In addition, ILE technology was harnessed to develop an in silico prediction system aimed at identifying chemicals with potential hERG inhibition liability. Our dataset consisted of 300 compounds classified as “active,” each with a half-maximal inhibitory concentration (IC50) of less than 10 µM. Additionally, we included 3000 compounds categorized as “non-active,” randomly selected from the ZINC database. Although hERG activity among some molecules in the non-active class was a possibility, their impact on the overall predictive accuracy of our model was considered minimal.

## 5. hERG Liability Indexing Model

A crucial aspect of our analysis was examining the diversity within the hERG blockers. Among the 300 compounds identified as hERG blockers, a significant majority (249 compounds) displayed a Tanimoto index of similarity below 0.7, indicating a high degree of chemical diversity. This diversity was critical for ensuring the robustness and generalizability of the prediction model.

Further extending this analysis to the proposed non-blockers from the ZINC database, we observed even greater diversity. An overwhelming 99.4% of these molecules had a Tanimoto similarity index below 0.7. This high level of diversity among non-blockers is particularly notable, as it underscores the wide-ranging applicability of our prediction system across various chemical entities.

**Figure 4:**
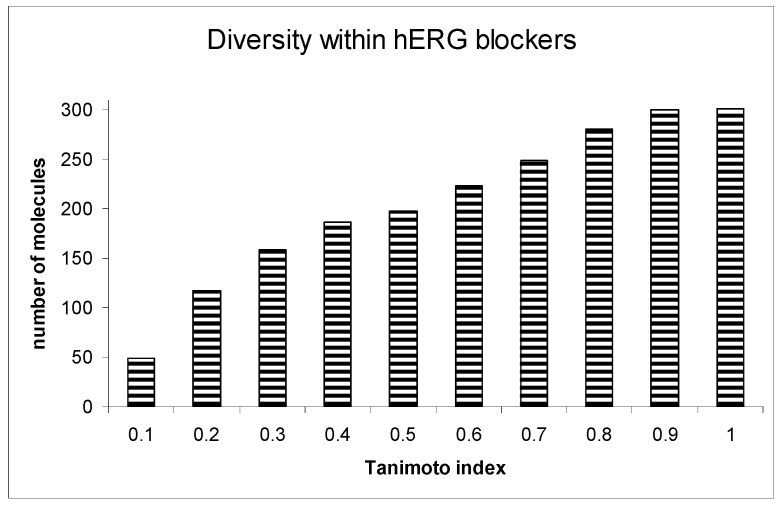
Diversity within hERG blockers.

**Figure 5:**
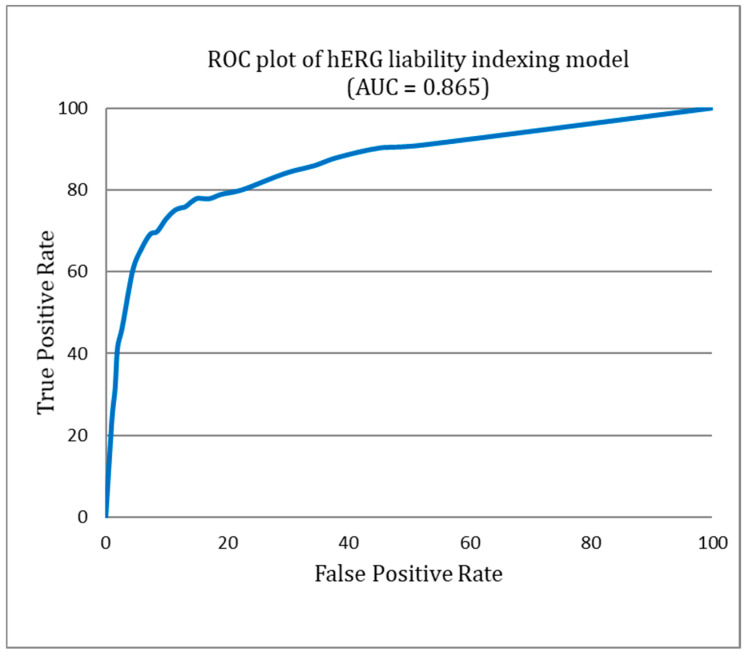
Receiver operating characteristic (ROC) curve of the hERG liability indexing model.

Figure 4 provides a detailed illustration of the diversity within both the hERG blockers and non-blockers. This visual representation illuminates the significant chemical variability encompassed by our study, a factor that plays a pivotal role in the robustness and efficacy of the ILE-based hERG inhibition liability prediction system. Figure 5 presents the receiver operating characteristic (ROC) curve for the indexing model of hERG blockers, with an area under the curve (AUC) of 0.865. This indicates that the model is highly accurate in distinguishing between hERG blockers and non-blockers.

**Figure 6:**
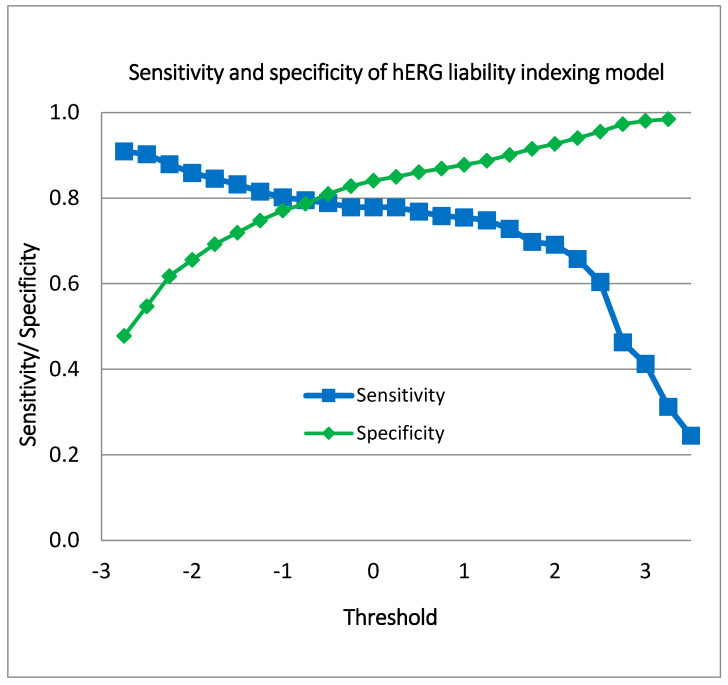
Sensitivity and specificity values of the indexing model for hERG liability.

Figure 6 illustrates the performance of the indexing model for hERG blockers, showcasing the range of sensitivity and specificity values achieved at various hERG liability thresholds. The sensitivity values span from 0.245 to 0.909, indicating that the model’s ability to correctly identify hERG blockers varies significantly with the threshold. Similarly, the specificity values range from 0.478 to 0.99, reflecting the model’s capability to accurately exclude non-blockers across different threshold settings.

**Figure 7:**
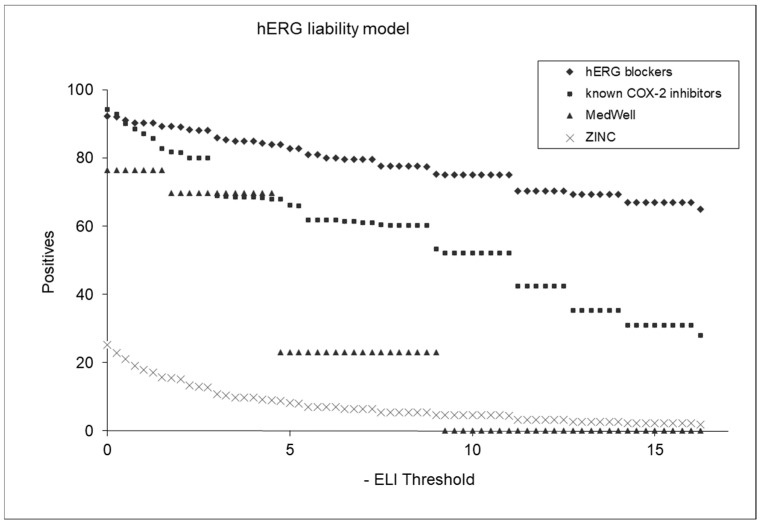
Comparative analysis of hERG liability across different compound classes.

Figure 7 displays plots of positive factors that underlie the hERG liability model’s predictions for various compound categories. The hERG liability index (ELI) is an index that indicates the likelihood of a molecule’s being hERG-liable. The higher the ELI value, the greater the chance that the molecule will bind to hERG and act on it. The plot features four distinct groups: hERG blockers, known COX-2 inhibitors, MedWell agents, and a selection of molecules randomly chosen from the ZINC database (referred to as “chemicals on the shelf”). Each group is represented by a different color or marker in the plot, allowing for a clear visual comparison of their hERG liability profiles. The hERG blockers are expected to show higher hERG liability scores, which serve as a benchmark for comparison. The known COX-2 inhibitors and MedWell agents are plotted to assess their relative hERG liabilities as predicted by the model, providing insights into their cardiac safety. The chemicals from the ZINC database offer a broader perspective, representing a diverse range of compounds. This comparative plot is useful for evaluating the effectiveness and discriminative power of the hERG liability model, illustrating its capability to differentiate between compounds with varying degrees of hERG inhibition.

### Model Validation and Performance Assessment

To rigorously assess the predictive performance of our models, we conducted a series of leave-1/3-out validation runs. In each run, a subset comprising 100 active and 1000 non-active compounds was randomly selected to serve as the holdout test set. This approach ensured a comprehensive evaluation across various subsets of the data.

Our results were encouraging; the models achieved a Matthews correlation coefficient (MCC) of approximately 0.8, based on the average of three independent test runs. This high MCC value indicates a strong correlation between the predicted and actual classifications and underscores the models’ robustness.

In terms of specific predictions, the models successfully identified nearly 89% of the hERG blockers (true positives), demonstrating their efficacy at detecting compounds with hERG liability. In addition, only about 10% of the molecules from the ZINC database were incorrectly classified as hERG blockers (false positives), showcasing the models’ precision.

Further analysis was performed by applying an ELI (hERG liability index) threshold of 5.0. This yielded a variety of identification percentages across different compound classes: 83% for hERG agents, 66% for COX-2 inhibitors, 23% for MedWell molecules, and 8.5% for ZINC chemicals potentially liable to hERG. These results highlight the models’ discriminative capability, effectively distinguishing between compounds with varying levels of hERG inhibition potential.

Overall, the validation and performance metrics demonstrate the high accuracy and reliability of our models for predicting hERG liability, affirming their potential utility in the drug discovery and development process.

## 6. Discussion

Accurate multiple sequence alignment (MSA) is essential for refining pairwise sequence alignments, reducing misalignments, and improving the construction of 3D models. Our method is particularly effective when analyzing protein families with a shared fold and multiple members, as it adeptly interprets the extensive data within sequence databases. It facilitates the precise alignment of multiple sequences and the development of superior comparative models.

To assess the performance of our method, we examined 124 unique proteins from the serine protease family, obtained from the Brookhaven Protein Data Bank (PDB). We calculated sequence identity scores for each sequence pair and employed the ILE method for optimal alignment. From the MSA, residues were identified in 98 proteins, while 28 proteins lacked coordinates for at least one residue in their 3D experimental structures. We extracted the alpha carbons (Cα) of these residues and performed structural superimpositions.

To evaluate the quality of our homology-based models, we compared the results with the proteins’ X-ray structures by measuring the Cα root-mean-square deviations (RMSDs). Table 5 presents a detailed overview of our method’s performance in homology modeling; it categorizes the results based on sequence identity ranges between the target and template sequences and reports the percentage of models that had RMSD values below the specified thresholds.

Furthermore, we processed the MSA matrix of the serine protease dataset, using our method to select sequence segments for comparative modeling. A voting approach was used, which determined each amino acid’s contribution to conservation at a sequence position, based on its frequency. This led to the definition of positional conservation thresholds (PCTs) to refine our models.

In conclusion, our method performed with exceptional accuracy on the MSA, resulting in highly reliable homology-based models. The integration of a voting-based approach and PCTs further enhanced the models’ accuracy. These findings highlight the effectiveness of the ILE method for addressing complex tasks in protein identification, classification, and structural modeling and extend its applicability beyond bioinformatics.

### Model for hERG-Toxic Molecules

In the realm of drug discovery, the ILE-based model emerges as a crucial tool for assessing the hERG liability of drug candidates. Its significance lies in its ability to inform decision-making processes, potentially leading to a notable reduction in attrition rates during drug development. By accurately predicting hERG toxicity, the model helps to identify risks early in the drug discovery pipeline, thereby streamlining the development process and reducing costly late-stage failures.

One of the key strengths of the ILE-based model is its utilization of 1D-2D descriptors. These descriptors not only effectively represent the necessary chemical properties for hERG liability prediction but are also advantageous in respect to the ease and speed of calculation. Using them enhances the model’s applicability in fast-paced research environments, where timely and efficient decision making is critical.

Furthermore, the simplicity and computational efficiency of the 1D-2D descriptors make the ILE model highly practical for widespread use in pharmaceutical research. They enable rapid screening of large libraries of compounds and early identification in the development cycle of those with hERG toxicity issues. This capability is particularly valuable in an industry where time and resource optimization are paramount.

The ILE model’s predictive power and practicality underscore its potential to become an integral part of the drug development toolkit. By providing researchers with a swift and reliable method for assessing hERG liability, the model can contribute significantly to the development of safer pharmaceuticals. Moreover, its integration into drug discovery pipelines will lead to a more streamlined, cost-effective, and ultimately successful drug development process.

Advantages and Limitations of ILE Technology: The ILE technology offers several significant advantages in the context of bioinformatics and drug discovery. One of its primary strengths is its exceptional accuracy in protein classification, as evidenced by its outstanding performance in classifying GPCRs in our study. The technology also excels in virtual high-throughput screening, efficiently identifying candidate molecules with desirable properties, and in homology-based modeling, where it has shown remarkable precision in creating 3D models. Furthermore, its straightforward design and processing efficiency make it a practical tool for rapid screening of chemicals for their bioactivity, significantly reducing the time and resources required to discover new hits in the drug discovery process.

However, despite these strengths, ILE technology does have certain limitations. A primary challenge is that, while the ILE model is highly effective within the specific contexts tested, its generalizability to other domains or different types of proteins may require further validation. Additionally, the reliance on predefined descriptors means the model’s performance can be constrained by the quality and relevance of the input data, potentially affecting its predictive accuracy in cases involving small or noisy datasets.

In conclusion, the ILE-based model for hERG-toxic molecules represents a significant advancement in drug safety assessment. Its ability to combine accuracy with practicality makes it a valuable asset to the pursuit of effective and safe therapeutic agents.

Future Perspectives: ILE technology presents promising potential for expanding its application beyond its current use. Future developments may include its integration into additional areas in the fields of bioinformatics, cheminformatics, and drug discovery. Specifically, the adaptability of the ILE algorithm could be harnessed to optimize processes in these fields, thereby enhancing the precision and efficiency of data analysis. We also plan to further refine the algorithmic processes of ILE, particularly to optimize the indexing chemicals for their bioactivity, potentially leading to even greater performance improvements. These advancements could establish ILE as a vital tool in various scientific endeavors, providing robust solutions to complex challenges in computational biology and drug discovery.

## 7. Conclusions

This paper introduces intelligent learning engine (ILE) optimization technology, a significant leap forward in the realm of bioinformatics and protein science. The ILE method, with its proficiency in multiple sequence alignment (MSA) and protein identification, classification, and structural modeling, effectively overcomes some of the most complex challenges in protein analysis. The empirical evaluation and results of this study, based on a dataset of 124 unique proteins from the serine protease family, validate the remarkable accuracy of the ILE method for MSA. Notably, its capability to identify residues in 98 proteins, even in the absence of experimental 3D structures, underscores its efficacy. Additionally, the method’s success in creating highly precise 3D models, as evidenced by Cα root-mean-square deviations (Cα RMSDs) compared with those observed in X-ray images, marks its significant potential for homology-based modeling. The incorporation of positional consensus templates (PCTs), developed through an innovative voting-based approach, considerably elevates the quality of these models.

The impact of the ILE method reaches beyond protein science, making a notable mark in the field of drug discovery. The technology’s advanced and discriminative model, utilizing 1D-2D descriptors for assessing hERG blockage by chemical entities, provides critical insights for developers of cardio-safe drug candidates. Particularly, the hERG toxicity indexing model is a key innovation, offering valuable data that can suggest a preference for certain types of molecules, for example, a preference for MedWell agents over COX-2 inhibitors in relation to cardio-safety.

The implications of this study are far-reaching, extending well beyond theoretical applications to practical uses across diverse scientific and technological sectors. By enhancing our understanding and manipulation of biological data and structures, ILE optimization technology lays the groundwork for significant advancements in areas such as drug discovery, healthcare, finance, and telecommunications. Its ability to improve screening accuracy across various domains stands as a testament to its transformative potential.

In conclusion, the ILE method adeptly addresses the intricate challenges of protein analysis and paves the way for novel applications in a plethora of fields. Its wide-ranging applicability and demonstrated effectiveness in both bioinformatics and other areas establish it as an indispensable tool in the continual pursuit of scientific and technological advancement.

In future work, we aim to explore the application of the simulated annealing algorithm, as reported by M. A. Rufal et al. [50], to improve the efficiency and accuracy of our ILE optimization technology. By integrating the principles of simulated annealing, we anticipate enhancing our algorithm’s ability to navigate complex optimization landscapes, potentially leading to even more significant breakthroughs in protein analysis, drug discovery, and beyond.

## Figures and Tables

**Table 1:** Comparison of the performance of established algorithms with the ILE method in the virtual high-throughput screening and identification of GPCR proteins.

Method	Prediction Accuracy in %
SVM dipeptide-based	99.5
SVM aa composition-based	96.5
BLAST	86.5
PROSITE pattern	92.0
Pfam profile HMMs	97.0
PRINTS	99.0
PROSITE profile using pfscan	97.0
QFC algorithm	99.5
Linear discriminant analysis	98.7
Quadratic discriminant analysis	98.5
Logistic discriminant analysis	97.7
K-nearest neighbor method (KNN)	98.3
ILE	100.0

**Table 2:** Performance of established algorithms for GPCR protein classification into superfamilies A, B, or C in virtual high-throughput screening, compared to the ILE method.

Method	Prediction Accuracy in %
SVM dipeptide-based	99.5
SVM aa composition-based	96.5
BLAST	86.5
PROSITE pattern	92.0
Pfam profile HMMs	97.0
PRINTS	99.0
PROSITE profile using pfscan	97.0
QFC algorithm	99.5
Linear discriminant analysis	98.7
Quadratic discriminant analysis	98.5
Logistic discriminant analysis	97.7
K-nearest neighbor method (KNN)	98.3
ILE	100.0

**Table 3:** Performance of established algorithms for GPCR protein classification at the first-subfamily level in virtual high-throughput screening, compared to the ILE method.

Method	Prediction Accuracy in %
SVM	88.4
BLAST	83.3
SAM-T2K HMM	69.9
kernNN	64.0
Decision tree	77.3
Naïve Bayes	93.0
ILE	99.8

**Table 4:** Performance of established algorithms for GPCR protein classification at the second-subfamily level in virtual high-throughput screening, compared to the ILE method.

Method	Prediction Accuracy in %
SVM	86.3
SVMtree	82.9
BLAST	74.5
SAM-T2K HMM	70.0
kernNN	51.0
Decision tree	70.8
Naïve Bayes	92.4
Covariant-discriminant	83.2
ILE	100.0

**Table 5:** Performance of the ILE method for homology modeling for different target–template identity classes within the serine protease family.

Percent Sequence Identity ^α^	Total Number of Models ^β^	Percent ^π^ Models with RMSD Lower Than 1 Å	Percent Models with RMSD Lower Than 2 Å	Percent Models with RMSD Lower Than 3 Å
25–29	15	40 ^Ω^	100	100
30–39	883	28	98	100
40–49	2365	50	99.9	100
50–59	423	75	100	100
60–69	51	90	100	100
70–79	181	100	100	100
80–89	289	100	100	100
90–95	44	100	100	100

^α^: Sequence identity range between the target and the template. ^β^: Total number of models in any given sequence identity range; the table summarizes 4251(1201) model template pairs. ^π^: The percent of models in a sequence identity range deviates by 1 Å or less from the corresponding experimental control structure; the following columns provide these percentages for other RMS deviations. ^Ω^: Secondary structure segments were used to generate models and evaluate the RMSDs in the performance of the ILE method, as tested on all 160 residues.

## Data Availability

All data generated or analyzed during this study are included within the manuscript.
